# Supplementary material for: Lack of TYK2 signaling enhances host resistance to Candida albicans skin infection
Source: Nat Commun. 2024 Dec 3;15:10493. doi: 10.1038/s41467-024-54888-6 (PMC11612186; doi:10.1038/s41467-024-54888-6)
Supplement: Supplementary file 1 — Supplementary Information [file 41467_2024_54888_MOESM1_ESM.pdf]

Supplementary information for:

Lack of TYK2 Signaling Enhances Host Resistance to *Candida albicans* Skin Infection

Sara Miranda[1], Caroline Lassnig[1,2], Kristina Schmidhofer[1], Hrönn Kjartansdottir[1], Claus Vogl[1], Simone Tangermann[3], Irina Tsymala[4,5], Verena Babl[1], Mathias Müller[1,2], Karl Kuchler[4,5] and Birgit Strobl[1, *]

Affiliations:

[1] Centre of Biological Sciences, University of Veterinary Medicine Vienna, Vienna, Austria

[2] Biomodels Austria, University of Veterinary Medicine Vienna, Vienna, Austria

[3] Centre of Pathobiology, University of Veterinary Medicine Vienna, Vienna, Austria

[4] Max Perutz Labs, Vienna Biocenter Campus (VBC), Vienna, Austria

[5] Medical University of Vienna, Center for Medical Biochemistry, Vienna, Austria

* Corresponding author

Email: birgit.strobl@vetmeduni.ac.at

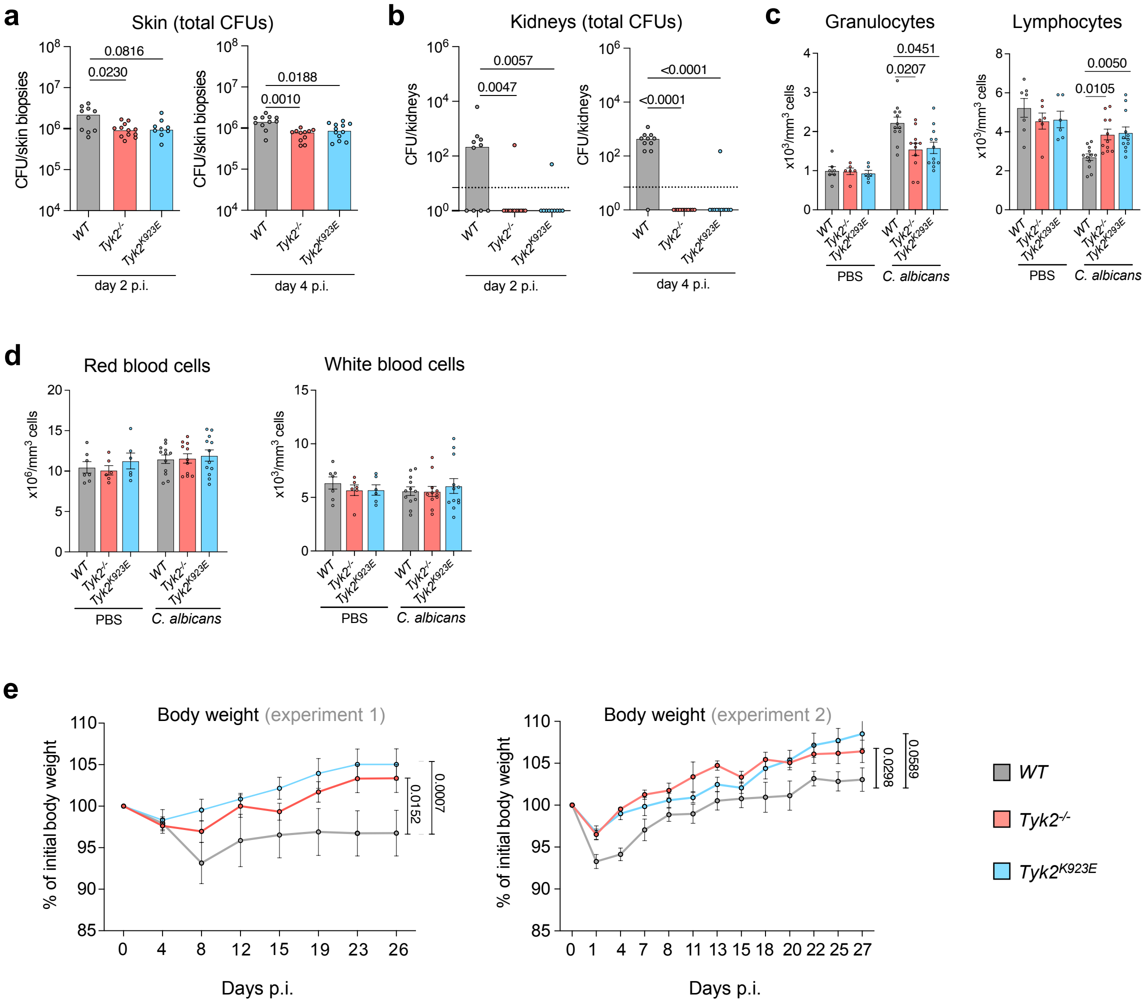

Supplementary Fig. S1. **a-b** Fungal load in the skin (**a**) and in the kidneys (**b**) was measured on days 2 and day 4 p.i. The total number of CFUs per skin (4 biopsies of 4 mm) and kidneys is represented. For each time-point, pooled data from 2 independent experiments are shown. Skin - day 2: n=10 *(WT)*, n=12 (*$Tyk2^{-/-}$*), n=9 *($Tyk2^{K923E}$)*; day 4: n=11 *(WT)*, n=11 (*$Tyk2^{-/-}$*), n=12 *($Tyk2^{K923E}$)*; Kidneys - day 2: n=10 *(WT)*, n=12 (*$Tyk2^{-/-}$*), n=10 *($Tyk2^{K923E}$)*; day 4: n=11 *(WT)*, n=11 (*$Tyk2^{-/-}$*), n=11 *($Tyk2^{K923E}$)*; Median values are given; n: biological replicates. The dotted line indicates the assay detection limit. **c-d** Blood cell composition on day 4 p.i was determined with a Vet ABC analyzer. The total numbers of granulocytes and lymphocytes (**c**) and of red and white blood cells (**d**) are shown. For each cell population, pooled data from 2 independent experiments are shown. PBS: n=7 (*WT*) and n=6 (*$Tyk2^{-/-}$* and *$Tyk2^{K923E}$*); *C. albicans*: n=12 *(WT* and *$Tyk2^{K923E}$)* and n=11 (*$Tyk2^{-/-}$*); Mean values ± SEM are given; n: biological replicates. **e** Body weight of infected *WT*, *$Tyk2^{-/-}$* and *$Tyk2^{K923E}$* mice was measured overtime and is shown as percentage relative to the weight on the day before the infection (set as 100%). Two independent experiments are separately represented. Experiment 1: n=6 (*WT* and *$Tyk2^{-/-}$*) and n=5 (*$Tyk2^{K923E}$*); Experiment 2: n=7 (*WT*) and n=6 (*$Tyk2^{-/-}$* and *$Tyk2^{K923E}$*); Mean values ± SEM are given; n: biological replicates. Statistical analysis was conducted using One-way ANOVA followed by Tukey's multiple comparison test (**a-e**). Statistical significance is only given for the comparison between the genotypes (**c,d**). Source data are provided as a Source Data file.

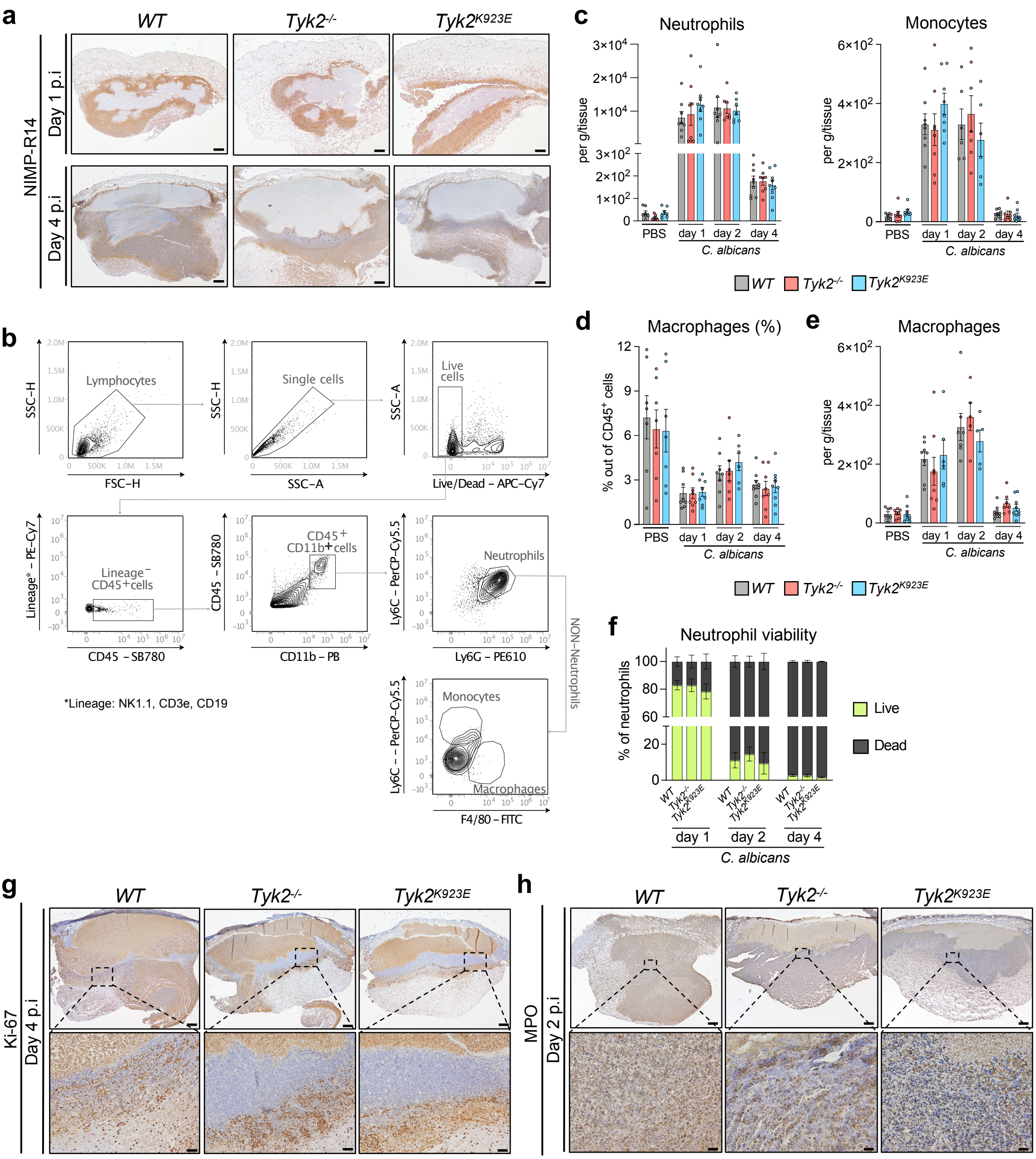

Supplementary Fig. S2. **a** Skin sections obtained from infected *WT, $Tyk2^{-/-}$* and *$Tyk2^{K923E}$* mice on day 1 and 4 p.i were stained with an anti-NIMP-R14 antibody. Data are representative of sections from 5 mice per genotype (day 1) and 4 *WT* and 5 *$Tyk2^{-/-}$* and *$Tyk2^{K923E}$* mice (day 4). Scale bar: 200 µm. **b** The gating strategy used to define skin-infiltrating neutrophils, monocytes and macrophages is shown. **c-e** Skin-infiltrating neutrophils (gated as CD45$^+$CD11b$^+$Ly6C$^+$Ly6G$^+$ cells) and monocytes (gated as CD45$^+$CD11b$^+$Ly6G$^-$Ly6C^{high}F4/80$^-$ cells) (**c**) and macrophages (gated as CD45$^+$CD11b$^+$Ly6G$^-$F4/80$^+$ cells) (**d, e**) were quantified by flow cytometry on days 1, 2 and 4 p.i. The number of these cells per gram of skin (**c, e**) or the percentage of cells out of myeloid CD45$^+$ CD11b$^+$ cells (**d**) is shown. For each time-point, pooled data from 2 independent experiments are shown. **c** PBS: n=7 (*WT, $Tyk2^{-/-}$, $Tyk2^{K923E}$*); *C. albicans* day 1: n=8 (*WT, $Tyk2^{-/-}$, $Tyk2^{K923E}$*); *C. albicans* day 2: n=8 (*WT)* and n=7 *($Tyk2^{-/-}$,$Tyk2^{K923E}$*); *C. albicans* day 4: n=9 (*WT, $Tyk2^{-/-}$)* and n=8 (*$Tyk2^{K923E}$*); **d** PBS: n=7 (*WT, $Tyk2^{-/-}$, $Tyk2^{K923E}$*); *C. albicans* day 1: n=8 (*WT, $Tyk2^{-/-}$, $Tyk2^{K923E}$*); *C. albicans* day 2: n=8 (*WT),* n=7 *($Tyk2^{-/-}$)* and n=6 (*$Tyk2^{K923E}$*); *C. albicans* day 4: n=9 (*WT, $Tyk2^{K923E}$)* and n=8 (*$Tyk2^{-/-}$*); **e** PBS: n=7 (*WT, $Tyk2^{-/-}$, $Tyk2^{K923E}$*); *C. albicans* day 1: n=8 (*WT, $Tyk2^{-/-}$*), n=6 (*$Tyk2^{K923E}$*); *C. albicans* day 2: n=7 (*WT),* n=5 *($Tyk2^{-/-}$,$Tyk2^{K923E}$*), day 4: n=9 (*WT, $Tyk2^{K923E}$)* and n=7 (*$Tyk2^{-/-}$*); Mean values ± SEM are given; n: biological replicates. **f** The viability of skin infiltrating neutrophils was quantified based on flow cytometry analysis with live/dead staining. For each time-point, pooled data from 2 experiments is shown. *C. albicans* day 1: n=8 (*WT, $Tyk2^{-/-}$,$Tyk2^{K923E}$*); *C. albicans* day 2: n=8 (*WT),* n=7 *($Tyk2^{-/-}$,$Tyk2^{K923E}$*); *C. albicans* day 4: n=9 (*WT, $Tyk2^{K923E}$)* and n=8 (*$Tyk2^{-/-}$*); Mean values ± SEM are given; n: biological replicates. **g** Skin sections obtained from infected *WT, $Tyk2^{-/-}$* and *$Tyk2^{K923E}$* mice on day 4 p.i were stained with an anti-Ki-67 antibody. Scale bar: 200 µm (top), 50 µm (bottom). **h** Skin sections obtained from infected *WT, $Tyk2^{-/-}$* and *$Tyk2^{K923E}$* mice on day 2 p.i were stained with an anti-MPO antibody. Scale bar: 200 µm (top) , 20 µm (bottom). Data are representative of sections from 4 *WT* and 5 *$Tyk2^{-/-}$* and *$Tyk2^{K923E}$* mice (**g**), and 7 mice per genotype (**h**). Statistical analysis was conducted using One-way ANOVA followed by Tukey's multiple comparison test (**c-f**). Statistical significance is only given for the comparison between the genotypes (**c-f**). Source data are provided as a Source Data file.

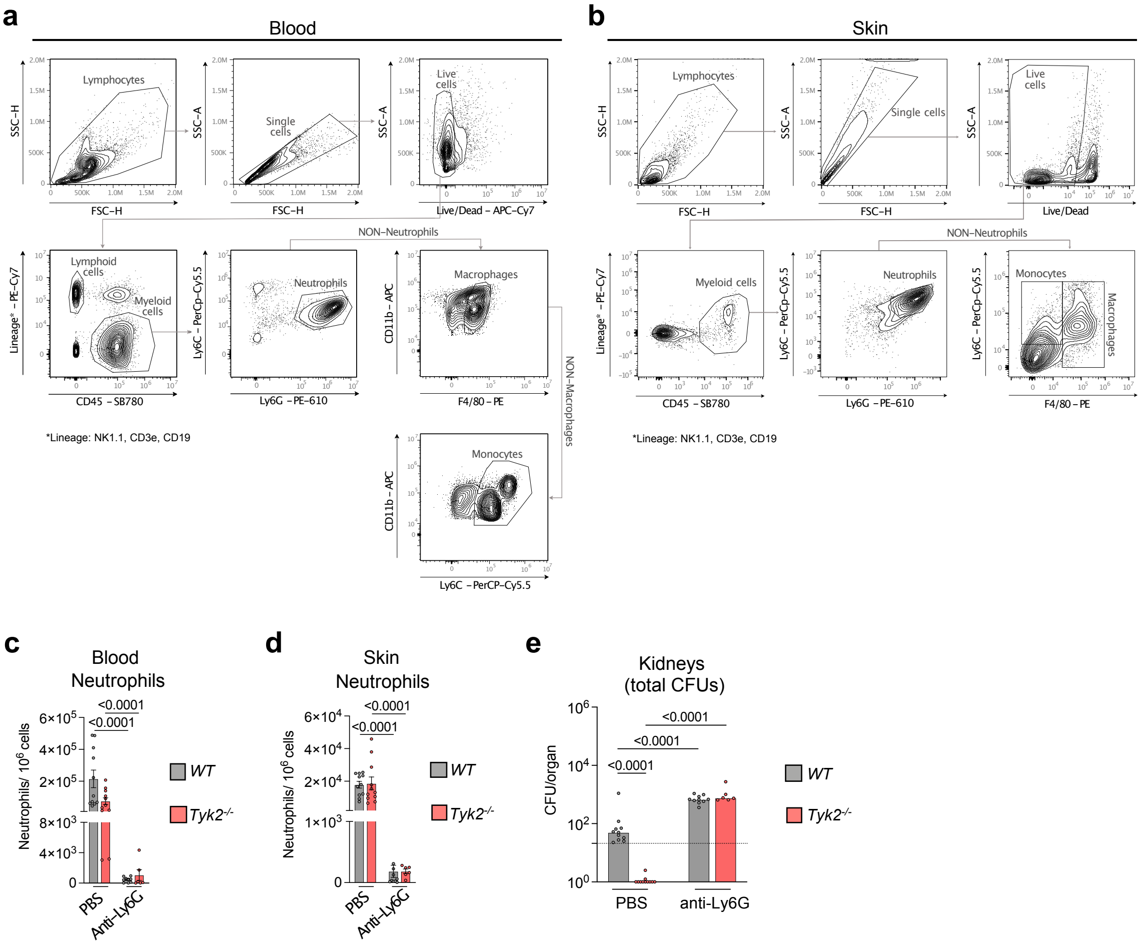

Supplementary Fig. S3. **a-b** The gating strategies used to define neutrophils, monocytes and macrophages in the blood (**a**) and the skin (**b**) are shown. **c-d** The numbers of neutrophils in the blood (**c**) and in the skin (**d**) are shown. Pooled data from 2 independent experiments are shown. **c** PBS: n=11 (*WT, Tyk2*$^{-/-}$); Anti-Ly6G: n=9 (*WT),* n=6 (*Tyk2*$^{-/-}$); **d** PBS: n=11 (*WT, Tyk2*$^{-/-}$); Anti-Ly6G: n=10 (*WT),* n=6 (*Tyk2*$^{-/-}$); Mean values ± SEM are given; n: biological replicates. **e** Fungal load in the kidneys of mice injected with anti-Ly6G or PBS was measured on day 2 p.i. PBS: n=11 (*WT, Tyk2*$^{-/-}$); Anti-Ly6G: n=11 (*WT),* n=6 (*Tyk2*$^{-/-}$); Mean values ± SEM are given; n: biological replicates. The dotted line indicates the assay detection limit. Statistical analysis was conducted using One-way ANOVA followed by Tukey's multiple comparison test (**c-e**). Source data are provided as a Source Data file.

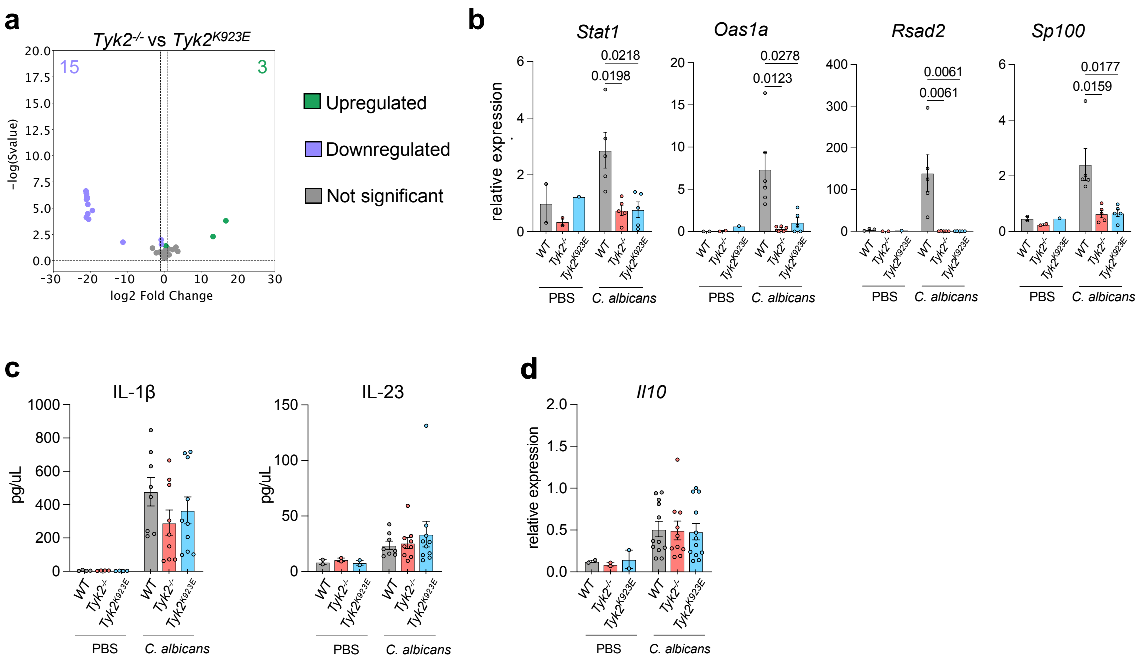

Supplementary Fig. S4. a Volcano plot showing the differentially expressed genes between *Tyk2*$^{-/-}$ and *Tyk2*^{K923E}. Genes were identified from DESeq2-normalized read counts of genes with a threshold of $p_{adj} < 0.05$ between *Tyk2*$^{-/-}$ and *Tyk2*^{K923E} cells. Data are from one experiment (n=3 per genotype). **b** mRNA levels of *Stat1, Oas1a, Rsad2,* and *Sp100* in the skin on day 1 p.i were measured by RT-qPCR. Data were normalized to the housekeeping gene *Ube2d2.* One experiment is shown. PBS: n=2 (*WT, Tyk2*$^{-/-}$), n=1 (*Tyk2*^{K923E}); *C. albicans:* n=5 (*WT, Tyk2*$^{-/-}$*, Tyk2*^{K923E}); Mean values ± SEM are given; n: biological replicates. **c** IL-1β and IL-23 in the skin on day 2 p.i were measured using a Luminex assay. Pooled data from 2 independent experiments are shown. IL-1β – PBS: n=4/genotype; *C. albicans,* n=8 (*WT*), n=10 (*Tyk2*$^{-/-}$), n=9 (*Tyk2*^{K923E}); IL-23 – PBS: n=2/genotype (PBS); *C. albicans:* n=8 (*WT*), n=10 (*Tyk2*$^{-/-}$), n=9 (*Tyk2*^{K923E}); Mean values ± SEM are given; n: biological replicates. **d** mRNA levels of *Il10* in the infected skin on day 4 p.i were measured by RT-qPCR. Data were normalized to the housekeeping gene *Ube2d2*. Pooled data from 2 independent experiments are shown. PBS: n=2 (PBS); *C. albicans:* n=12 (*WT*), n=10 (*Tyk2*$^{-/-}$), n=12 (*Tyk2*^{K923E}); Mean values ± SEM are given; n: biological replicates. Statistical analysis was conducted using One-way ANOVA followed by Tukey's multiple comparison test (**b-d**). Statistical significance is only given for the comparison between the genotypes (**b-d**). Source data are provided as a Source Data file.

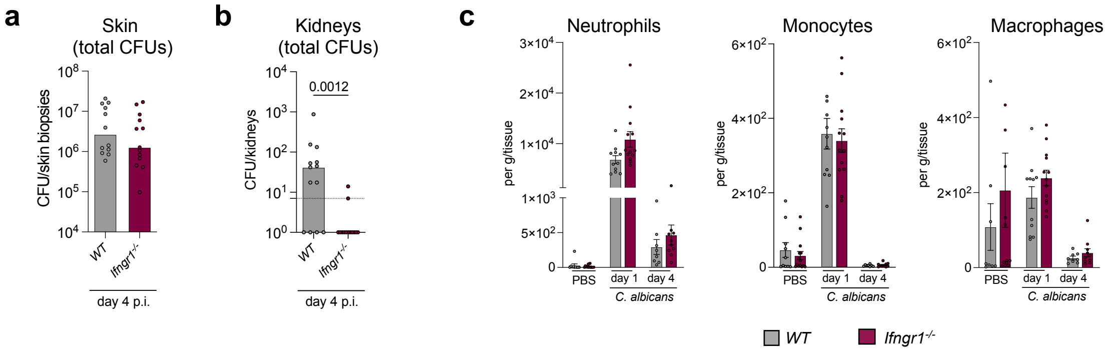

Supplementary Fig. S5. a-b Fungal load in the skin (**a**) and in the kidneys (**b**) was measured on day 4 p.i. The total number of CFUs per skin (4 biopsies of 4 mm) and kidneys is represented. Pooled data from 2 independent experiments are shown. **a** n=12 *(WT),* n=13 *(Ifngr1$^{-/-}$);* **b** n=13 *(WT, Ifngr1$^{-/-}$)*; Median values are given; n: biological replicates. The dotted line indicates the assay detection limit. **c** Skin-infiltrating neutrophils (gated as CD45$^+$CD11b$^+$Ly6C$^+$Ly6G$^+$ cells), monocytes (gated as CD45$^+$CD11b$^+$Ly6G$^-$Ly6C^{high}F4/80$^-$ cells) and macrophages (gated as CD45$^+$CD11b$^+$Ly6G$^-$F4/80$^+$ cells) were quantified based on flow cytometry analysis on day 1 and 4 p.i. For each time-point, pooled data from 2 independent experiments are shown. Neutrophils and Monocytes – PBS: n=10 (*WT*, *Ifngr1$^{-/-}$*); *C. albicans* day 1: n=11 *(WT),* n=13 (*Ifngr1$^{-/-}$*); *C. albicans* day 4: n=8 *(WT),* n=10 (*Ifngr1$^{-/-}$*); Macrophages – PBS: n=8 (*WT*), n=13 *(Ifngr1$^{-/-}$)*; *C. albicans* day 1: n=11 *(WT),* n=13 (*Ifngr1$^{-/-}$*); *C. albicans* day 4: n=8 *(WT),* n=10 (*Ifngr1$^{-/-}$*); Mean values ± SEM are given; n: biological replicates. Statistical analysis was conducted using a two-tailed Mann Whitney test (**a, b**) and One-way ANOVA followed by Tukey's multiple comparison test (**c**). Statistical significance is only given for the comparison between the genotypes (**c**). Source data are provided as a Source Data file.

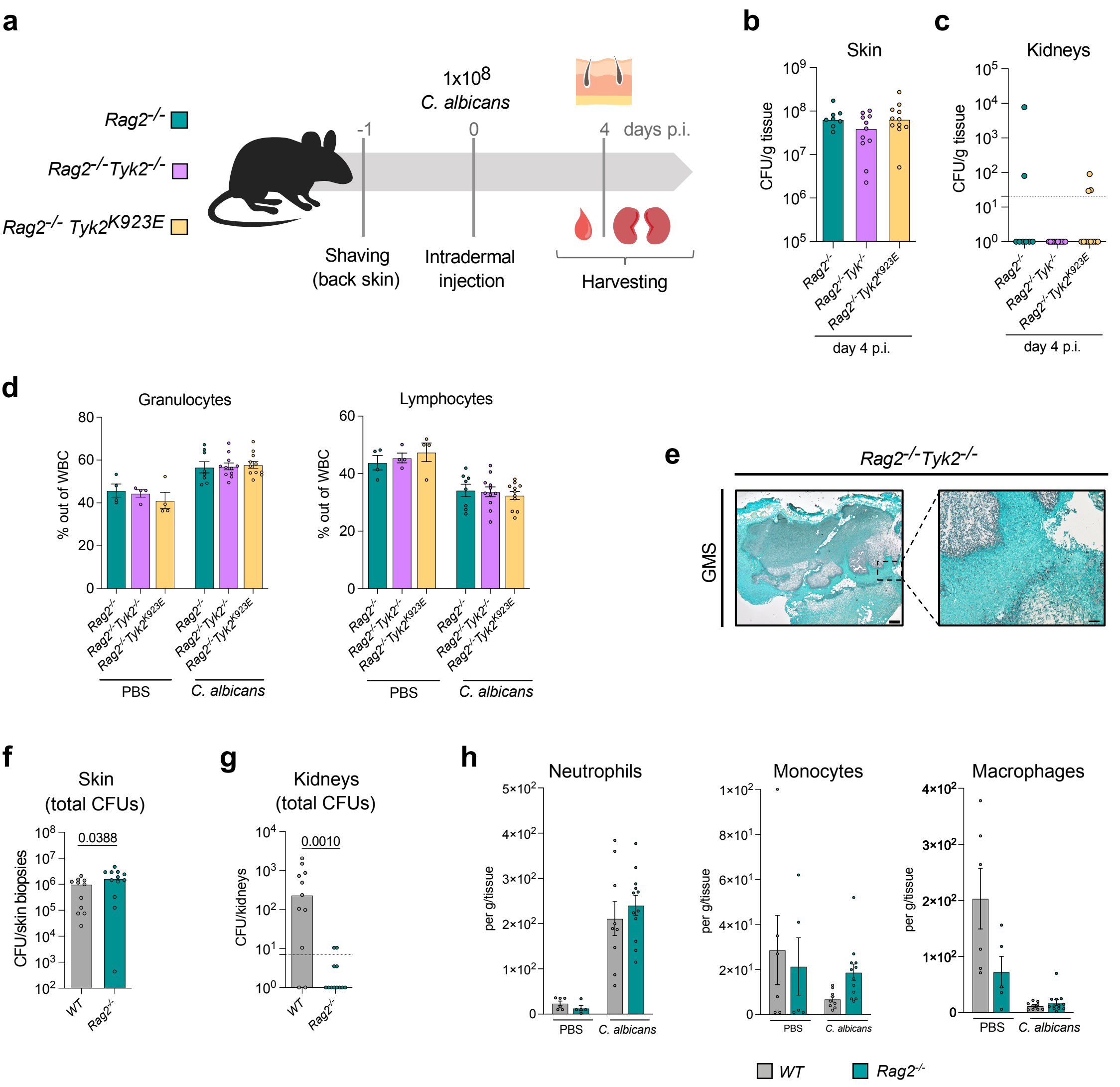

Supplementary Fig. S6. a *$Rag2^{-/-}$*, *$Rag2^{-/-}Tyk2^{-/-}$* and *$Rag2^{-/-}Tyk2^{K923E}$* mice were infected as described in the legend to Fig. 1. **b-c** Fungal load in the skin (**b**) and kidneys (**c**) was measured on day 4 p.i. Pooled data from 2 independent experiments are shown. n=8 *($Rag2^{-/-}$)*, n=11 *($Rag2^{-/-}Tyk2^{-/-}$, $Rag2^{-/-}Tyk2^{K923E}$)*; Median values are given; n: biological replicates. The dotted line indicates the assay detection limit. **d** Blood cell composition on day 4 p.i was determined with a Vet ABC analyzer. The percentage of granulocytes and lymphocytes out of total white blood cells (WBC) is shown. For each cell population, pooled data from 2 independent experiments are shown. PBS: n=4 *($Rag2^{-/-}$, $Rag2^{-/-}Tyk2^{-/-}$, $Rag2^{-/-}Tyk2^{K923E}$); C. albicans:* n=8 (*$Rag2^{-/-}$*), n=11 (*$Rag2^{-/-}Tyk2^{-/-}$*, *$Rag2^{-/-}Tyk2^{K923E}$*); Mean values ± SEM are given; n: biological replicates. **e** Representative pictures of the infected skin on day 4 p.i. A GMS staining of the skin sections is shown. Data are representative of sections from 4 mice per genotype. Scale bar represents 200 µm (left) or 50 µm (right). **f-g** Fungal load in the skin (**f**) and in the kidneys (**g**) was measured on day 4 p.i. The total number of CFUs per skin (4 biopsies of 4 mm) and kidneys and pooled data from 2 independent experiments are shown. n=11 *(WT)*, n=12 *($Rag2^{-/-}$)*; Median values are given; n: biological replicates. The dotted line indicates the assay detection limit. **h** Skin-infiltrating neutrophils (gated as $CD45^+CD11b^+Ly6C^+Ly6G^+$ cells), monocytes (gated as $CD45^+CD11b^+Ly6G^-Ly6C^{high}F4/80^-$ cells) and macrophages (gated as $CD45^+CD11b^+Ly6G^-F4/80^+$ cells) were quantified based on flow cytometry analysis on day 4 p.i. Pooled data from 2 independent experiments are shown. PBS: n=6 (*WT*), n=5 (*$Rag2^{-/-}$*); *C. albicans:* n=9 *(WT)*, n=12 (*$Rag2^{-/-}$*); Mean values ± SEM are given; n: biological replicates. Statistical analysis was conducted using One-way ANOVA followed by Tukey's multiple comparison test (**b-d, h**) or two-tailed Mann Whitney test (**f-g**). Statistical significance is only given for the comparison between the genotypes (**b-d, h**). Source data are provided as a Source Data file.

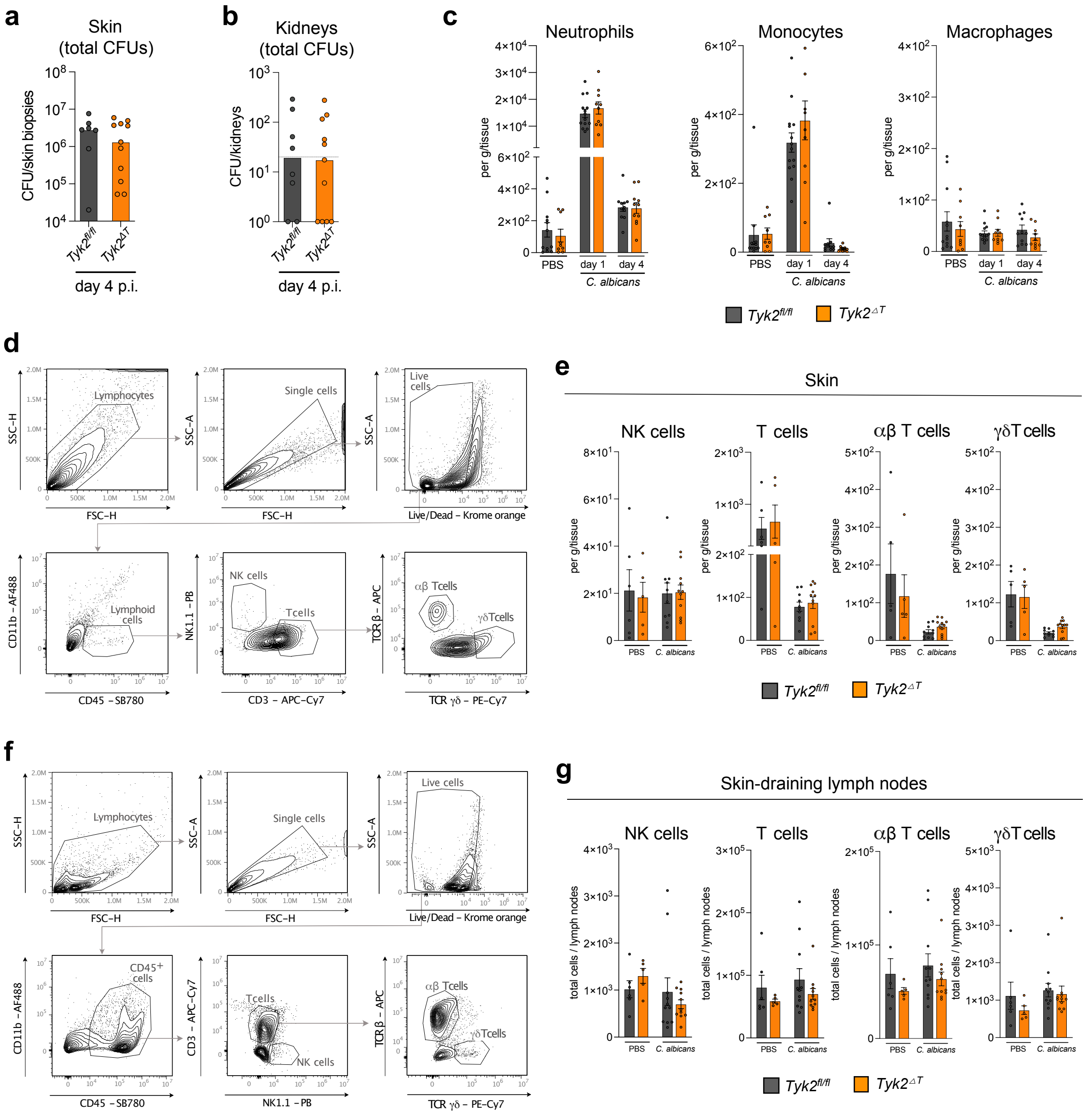

Supplementary Fig. S7. a-b Fungal load in the skin (**a**) and in the kidneys (**b**) was measured on day 4 p.i. The total number of CFUs per skin (4 biopsies of 4 mm) and kidneys is represented. Pooled data from 2 independent experiments are shown. **a** n=7 (*$Tyk2^{fl/fl}$)*, n=11 (*$Tyk2^{\Delta T}$*); **b** n=8 (*$Tyk2^{fl/fl}$)*, n=11 (*$Tyk2^{\Delta T}$);* Median values are given; n: biological replicates. The dotted line indicates the assay detection limit. **c** Skin-infiltrating neutrophils (gated as CD45$^+$CD11b$^+$Ly6C$^+$Ly6G$^+$ cells), monocytes (gated as CD45$^+$CD11b$^+$Ly6G$^-$Ly6C^{high}F4/80$^-$ cells) and macrophages (gated as CD45$^+$CD11b$^+$Ly6G$^-$F4/80$^+$ cells) were quantified by flow cytometry analysis on 4 p.i. Pooled data from 2 independent experiments are shown. PBS: n=12 (*$Tyk2^{fl/fl}$)*, n=9 (*$Tyk2^{\Delta T}$*); *C. albicans* day 1: n=14 (*$Tyk2^{fl/fl}$), n=10 ($Tyk2^{\Delta T}$); C. albicans* day 4: n=11 (*$Tyk2^{fl/fl}$)*, n=11 (*$Tyk2^{\Delta T}$*, Neutrophils), n=10 (*$Tyk2^{\Delta T}$*, Monocytes, Macrophages); Mean values ± SEM are given; n: biological replicates. **d-g** The gating strategies used to identify NK cells, T cells, M M T cells and M M T cells in the skin (**d**) and in the skin-draining lymph nodes (**f**) on day 4 p.i. and the total numbers of each of these populations per gram of skin (**e**) or in the lymph nodes (**g**) are presented. Pooled data from 2 independent experiments are shown. **e** PBS: n=6 (*$Tyk2^{fl/fl}$, NK cells and T cells)*, n=5 (*$Tyk2^{fl/fl}$, M M T cells and M M T cells)*, n=5 (*$Tyk2^{\Delta T}$*); *C. albicans:* n=10 (*$Tyk2^{fl/fl}$)*, n=11 (*$Tyk2^{\Delta T}$*); **g** PBS: n=6 (*$Tyk2^{fl/fl}$)*, n=5 (*$Tyk2^{\Delta T}$*); *C. albicans:* n=11 (*$Tyk2^{fl/fl}$, $Tyk2^{\Delta T}$*); Mean values ± SEM are given; n: biological replicates. Statistical analysis was conducted using two-tailed Mann Whitney test (**a,b**) or One-way ANOVA followed by Tukey's multiple comparison test (**c, e, g**). Statistical significance is only given for the comparison between the genotypes (**c, e, g**). Source data are provided as a Source Data file.

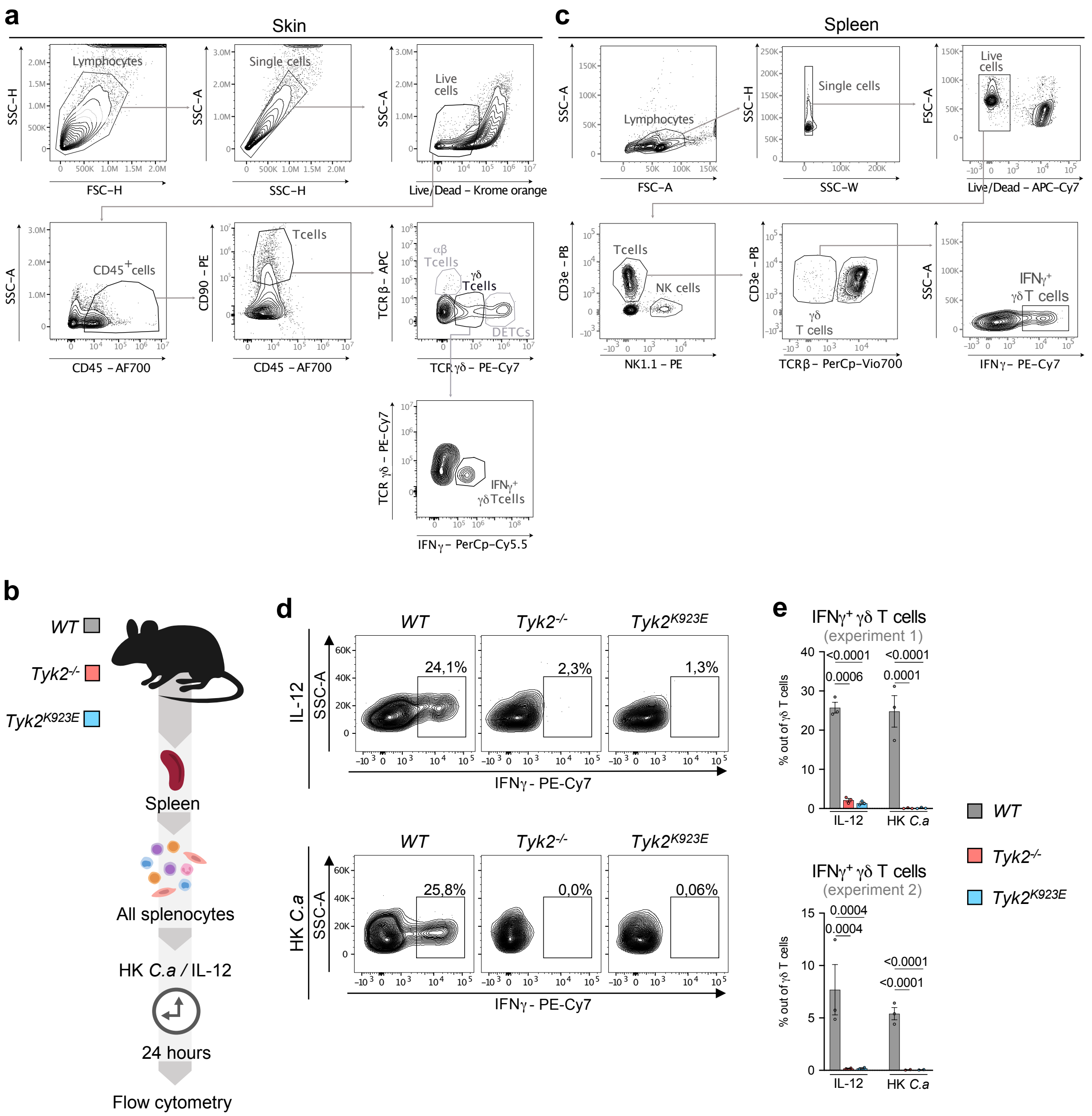

Supplementary Fig. S8. a The gating strategy used to identify IFNγ⁺ γδ T cells out of skin cells incubated with heat-killed *C. albicans,* IL-2 or IL-12 is shown. **b** The spleens of *WT, Tyk2*$^{-/-}$ and *Tyk2*^{K923E} mice were collected and single cell suspensions were prepared. 1x10^{6} splenocytes were incubated for 24 hours with heat-killed *C. albicans* (HK *C.a)* or IL-12 and stained for flow cytometry analysis. **c** The gating strategy used to identify IFNγ⁺ γδ T cells out of splenocytes is shown. **d-e** The percentage of IFNγ⁺ cells out of γδ T cells was determined by flow cytometry analysis. Representative contour plots are presented (**d**) and two independent experiments are shown (**e**). Experiment 1: n=3 (*WT, Tyk2*$^{-/-}$*, Tyk2*^{K923E}*);* Experiment 2: n=3 (*WT*), n=2 (*Tyk2*$^{-/-}$*, Tyk2*^{K923E}); Mean values ± SEM are given; n: biological replicate; each biological replicate was plated in duplicates. Statistical analysis was conducted using One-way ANOVA followed by Tukey's multiple comparison test. Statistical significance is only given for the comparison between the genotypes. Source data are provided as a Source Data file.
