# Supplementary material for: Lack of TYK2 signaling enhances host resistance to Candida albicans skin infection
Source: Nat Commun. 2024 Dec 3;15:10493. doi: 10.1038/s41467-024-54888-6 (PMC11612186; doi:10.1038/s41467-024-54888-6)
Supplement: Supplementary file 2 — Description of Additional Supplementary Files [file 41467_2024_54888_MOESM2_ESM.pdf]

Description of Additional Supplementary Files

File Name: Supplementary Data
Description: Differentially expressed genes between WT, Tyk2-/- and Tyk2K923E skin-infiltrating myeloid cells.
