# Supplementary material for: Lack of TYK2 signaling enhances host resistance to Candida albicans skin infection
Source: Nat Commun. 2024 Dec 3;15:10493. doi: 10.1038/s41467-024-54888-6 (PMC11612186; doi:10.1038/s41467-024-54888-6)
Supplement: Supplementary file 4 — Reporting Summary [file 41467_2024_54888_MOESM4_ESM.pdf]

nature portfolio

Corresponding author(s): Birgit Strobl

Last updated by author(s): Nov 12, 2024

Reporting Summary

Nature Portfolio wishes to improve the reproducibility of the work that we publish. This form provides structure for consistency and transparency in reporting. For further information on Nature Portfolio policies, see our Editorial Policies and the Editorial Policy Checklist.

Please do not complete any field with "not applicable" or n/a. Refer to the help text for what text to use if an item is not relevant to your study.
For final submission: please carefully check your responses for accuracy; you will not be able to make changes later.

Statistics

For all statistical analyses, confirm that the following items are present in the figure legend, table legend, main text, or Methods section.

n/a	Confirmed	
☐	☒	The exact sample size (*n*) for each experimental group/condition, given as a discrete number and unit of measurement
☐	☒	A statement on whether measurements were taken from distinct samples or whether the same sample was measured repeatedly
☐	☒	The statistical test(s) used AND whether they are one- or two-sided<br>*Only common tests should be described solely by name; describe more complex techniques in the Methods section.*
☐	☒	A description of all covariates tested
☐	☒	A description of any assumptions or corrections, such as tests of normality and adjustment for multiple comparisons
☐	☒	A full description of the statistical parameters including central tendency (e.g. means) or other basic estimates (e.g. regression coefficient) AND variation (e.g. standard deviation) or associated estimates of uncertainty (e.g. confidence intervals)
☐	☒	For null hypothesis testing, the test statistic (e.g. *F*, *t*, *r*) with confidence intervals, effect sizes, degrees of freedom and *P* value noted<br>*Give P values as exact values whenever suitable.*
☒	☐	For Bayesian analysis, information on the choice of priors and Markov chain Monte Carlo settings
☒	☐	For hierarchical and complex designs, identification of the appropriate level for tests and full reporting of outcomes
☒	☐	Estimates of effect sizes (e.g. Cohen's *d*, Pearson's *r*), indicating how they were calculated

Our web collection on statistics for biologists contains articles on many of the points above.

Software and code

Policy information about availability of computer code

Data collection: Flow cytometry data was acquired using a BD FACSCanto II or a CytoFLEX flow cytometer
Cytokine levels (Luminex assays) were measured on a Bio-Plex Reader (Bio-Rad).

Data analysis

For manuscripts utilizing custom algorithms or software that are central to the research but not yet described in published literature, software must be made available to editors and reviewers. We strongly encourage code deposition in a community repository (e.g. GitHub). See the Nature Portfolio guidelines for submitting code & software for further information.

Flow cytometry data were analysed with CytExpert and FlowJo.
GraphPad Prism Version 10 was used to plot bar graphs and to perform experimental statistical analysis.
RNA Seq data analysis: Acquired NGS reads were aligned to the Genome Reference Consortium GRCm39 assembly using Spliced Transcripts Alignment to a Reference (STAR, v2.7.3a), with the Ensembl transcript annotation from version e105 serving as reference transcriptome. Aligned reads were assigned to genomic features using featureCounts (v2.0.0); differential expression analysis was done with RStudio (http://www.rstudio.com/) (v2023.03.1-446) Bioconductor (v3.15) DESEq2 (v1.36.0) package based on a negative binomial distributed model. The heatmap displaying z-scores for differentially expressed genes was generated using the Python (v3.9.13) data visualization libraries matplotlib (v3.7.1) and seaborn (v0.11.2) Respective z-scores were calculated using the Python sklearn (v1.2.1) library. Pathway analysis of the differentially expressed genes was carried out using Reactome (v86) an open-source, curated and peer-reviewed pathway database.

Data

Policy information about availability of data

All manuscripts must include a data availability statement. This statement should provide the following information, where applicable:

- Accession codes, unique identifiers, or web links for publicly available datasets
- A description of any restrictions on data availability
- For clinical datasets or third party data, please ensure that the statement adheres to our policy

RNA-seq data has been deposited in the NCBI GEO repository under accession number GSE278915. Source data are provided with this paper.

Research involving human participants, their data, or biological material

Policy information about studies with human participants or human data. See also policy information about sex, gender (identity/presentation), and sexual orientation and race, ethnicity and racism.

Reporting on sex and gender	*Use the terms sex (biological attribute) and gender (shaped by social and cultural circumstances) carefully in order to avoid confusing both terms. Indicate if findings apply to only one sex or gender; describe whether sex and gender were considered in study design, whether sex and/or gender was determined based on self-reporting or assigned and methods used. Provide in the source data disaggregated sex and gender data, where this information has been collected, and if consent has been obtained for sharing of individual-level data, provide overall numbers in this Reporting Summary. Please state if this information has not been collected. Report sex- and gender-based analyses where performed, justify reasons for lack of sex- and gender-based analysis.*
Reporting on race, ethnicity, or other socially relevant groupings	*Please specify the socially constructed or socially relevant categorization variable(s) used in your manuscript and explain why they were used. Please note that such variables should not be used as proxies for other socially constructed/relevant variables (for example, race or ethnicity should not be used as a proxy for socioeconomic status). Provide clear definitions of the relevant terms used, how they were provided (by the participants/respondents, the researchers, or third parties), and the method(s) used to classify people into the different categories (e.g. self-report, census or administrative data, social media data, etc.) Please provide details about how you controlled for confounding variables in your analyses.*
Population characteristics	*Describe the covariate-relevant population characteristics of the human research participants (e.g. age, genotypic information, past and current diagnosis and treatment categories). If you filled out the behavioural & social sciences study design questions and have nothing to add here, write "See above."*
Recruitment	*Describe how participants were recruited. Outline any potential self-selection bias or other biases that may be present and how these are likely to impact results.*
Ethics oversight	*Identify the organization(s) that approved the study protocol.*

Note that full information on the approval of the study protocol must also be provided in the manuscript.

Field-specific reporting

Please select the one below that is the best fit for your research. If you are not sure, read the appropriate sections before making your selection.

☒ Life sciences ☐ Behavioural & social sciences ☐ Ecological, evolutionary & environmental sciences

For a reference copy of the document with all sections, see nature.com/documents/nr-reporting-summary-flat.pdf

Life sciences study design

All studies must disclose on these points even when the disclosure is negative.

Sample size	Sample size was chosen based on our previous studies (i.e. experience with the experimental model/ and the specific type of analysis). Differences in sample size between mice of the different genotypes in some experiments is due to differences in availability of age- and sex-matched mice. All experiments were done twice independently to ensure reproducibility of results. RNA-seq data were from 3 mice / genotype.
Data exclusions	Data from in vivo experiments were only excluded when several parameters indicated non-responsiveness to the infection or when there was a clear technical error; no data were excluded from in vitro studies.
Replication	Experimental findings were reproducible across 2 independent experiments.
Randomization	Experimental groups were not randomized. Age- and sex-matched animals were used for experiments.
Blinding	Histopathological analysis was performed in a blinded manner. Blinding was not necessary for all other types of analyses as they did not involve subjective interpretation.

Reporting for specific materials, systems and methods

We require information from authors about some types of materials, experimental systems and methods used in many studies. Here, indicate whether each material, system or method listed is relevant to your study. If you are not sure if a list item applies to your research, read the appropriate section before selecting a response.

Materials & experimental systems

n/a	Involved in the study
☐	☒ Antibodies
☐	☐ Eukaryotic cell lines
☐	☐ Palaeontology and archaeology
☐	☒ Animals and other organisms
☐	☐ Clinical data
☐	☐ Dual use research of concern
☐	☐ Plants

Methods

n/a	Involved in the study
☐	☐ ChIP-seq
☐	☒ Flow cytometry
☐	☐ MRI-based neuroimaging

Antibodies

Antibodies used

For IHC, following antibodies were used: NIMP-R14 (ab2557; Abcam, Cambridge, United Kingdom), anti-Ki-67 (14-5698-82; eBioscience/Thermofisher) and anti-MPO (AF3667-SP; Bio-techne, Minneapolis, USA); secondary antibodies: goat anti-rat IgG:Biotin (STAR131B; Bio-Rad) and swine anti-rabbit (E0353; DAKO-Agilent, Santa Clara, California, USA).
For flow cytometry following antibodies, all from Thermofisher, were used: anti CD16/CD32 (14-0161-85), PE anti-F4/80 (12-4801-82), FITC anti F4/80 (11-4801-82), PE-610 anti-Ly-6G (61-9668-80), PerCp-Cy5.5 anti-Ly-6C (45-5932-80), PE-Cy7 anti-CD3e (25-0031-82), PB anti-CD3e (48-0033-82), APC-eF780 anti-CD3e (47-0031-82), SB600 anti-CD4 (63-0041-80), AF700 anti-CD8a (56-0081-82), PE anti-NK1.1 (12-5941-82), PB anti-NK1.1 (48-5941-82), PE-Cy7 anti-NK1.1 (25-5941-82), PE-Cy7 anti-CD19 (25-0193-82), AF700 anti-CD11c (56-0114-82), PB anti CD11b (48-0112-82), APC anti-CD11b (17-0112-82), AF488 anti-CD11b (53-0112-82), PE anti-CD90.2 (12-0902-81), SB780 anti-CD45 (78-0451-82), PerCp-Cy5.5 anti-IFNg(45-7311-82), PE-Cy7 anti-IFNg (25-7311-82). In addition, AF700 anti-CD45 (103128; Biolegend), PE-Cy7 anti-TCRgd (118124; Biolegend), APC anti-TCRβ (109212; Biolegend), PerCp-Vio700 anti-TCRβ (130-120-827; Miltenyi, Bergisch Gladbach, Germany).
The following antibodies, all from Thermofisher, were used for FACS-sorting: anti CD16/CD32 (14-0161-85), PerCp-Cy5.5 anti-CD3e (45-0031-82), PerCp-Cy5.5 anti-NKp46 (46-3351-82), PerCp-Cy5.5 anti-CD19 (45-0193-82), and PE anti-CD45 (12-0451-83).
For neutrophil depletion: anti-Ly6G (Ab00295-2.0; Absolute antibody, Cleveland, United Kingdom).

Validation

The antibody validation is provided on supplier website.

Eukaryotic cell lines

Policy information about cell lines and Sex and Gender in Research

Cell line source(s)

State the source of each cell line used and the sex of all primary cell lines and cells derived from human participants or vertebrate models.

Authentication

Describe the authentication procedures for each cell line used OR declare that none of the cell lines used were authenticated.

Mycoplasma contamination

Confirm that all cell lines tested negative for mycoplasma contamination OR describe the results of the testing for mycoplasma contamination OR declare that the cell lines were not tested for mycoplasma contamination.

Commonly misidentified lines
(See ICLAC register)

Name any commonly misidentified cell lines used in the study and provide a rationale for their use.

Palaeontology and Archaeology

Specimen provenance

Provide provenance information for specimens and describe permits that were obtained for the work (including the name of the issuing authority, the date of issue, and any identifying information). Permits should encompass collection and, where applicable, export.

Specimen deposition

Indicate where the specimens have been deposited to permit free access by other researchers.

Dating methods

If new dates are provided, describe how they were obtained (e.g. collection, storage, sample pretreatment and measurement), where they were obtained (i.e. lab name), the calibration program and the protocol for quality assurance OR state that no new dates are provided.

☐ Tick this box to confirm that the raw and calibrated dates are available in the paper or in Supplementary Information.

Ethics oversight | *Identify the organization(s) that approved or provided guidance on the study protocol, OR state that no ethical approval or guidance was required and explain why not.*

Note that full information on the approval of the study protocol must also be provided in the manuscript.

Animals and other research organisms

Policy information about studies involving animals; ARRIVE guidelines recommended for reporting animal research, and Sex and Gender in Research

Laboratory animals	All mice used in this study were bred under specific-pathogen-free conditions according to Federation of European Laboratory Animal Science Associations (FELASA) guidelines.<br>Wild-type (WT, C57BL/6N), Tyk2-/-, Tyk2K923E, Tyk2fl/fl and Ifngr1-/- mice were previously described To generate mice that lack TYK2 in alphabeta-T cells (Tyk2deltaT), Tyk2fl/fl mice were crossed to CD4-cre mice(B6.Cg-Tg(Cd4-cre)1Cwi, kindly provided by Wilfried Ellmeier, Medical University of Vienna). Rag2 -/- (B6.129S6-Rag2tm1Fw) mice were kindly provided by Veronika Sexl, University of Veterinary Medicine Vienna References for mouse strains are given in the manuscript Age- and sex-matched (8-12 weeks) mice were used for all experiments.
Wild animals	The study did not involve wild animals
Reporting on sex	Female and male mice were used in this study.
Field-collected samples	No field-collected samples are included.
Ethics oversight	All mice used in this study were bred at the University of Veterinary Medicine Vienna under specific-pathogen-free conditions according to Federation of European Laboratory Animal Science Associations (FELASA) guidelines. All animal experiments were approved by the ethics and animal welfare committee of the University of Veterinary Medicine Vienna and the Austrian Federal Ministry of Science and Research according to §§ 26ff of Animal Experiments Act, Tierversuchsgesetz 2012—TVG 2012 (BMWFW-68.205/0032- WF/II/3b/2014 and BMBWF-68.205/0173-V/3b/2019) and conform to the guidelines of FELASA and ARRIVE (Animal Research: Reporting of In Vivo Experiments).

Note that full information on the approval of the study protocol must also be provided in the manuscript.

Clinical data

Policy information about clinical studies

All manuscripts should comply with the ICMJE guidelines for publication of clinical research and a completed CONSORT checklist must be included with all submissions.

Clinical trial registration	*Provide the trial registration number from ClinicalTrials.gov or an equivalent agency.*
Study protocol	*Note where the full trial protocol can be accessed OR if not available, explain why.*
Data collection	*Describe the settings and locales of data collection, noting the time periods of recruitment and data collection.*
Outcomes	*Describe how you pre-defined primary and secondary outcome measures and how you assessed these measures.*

Dual use research of concern

Policy information about dual use research of concern

Hazards

Could the accidental, deliberate or reckless misuse of agents or technologies generated in the work, or the application of information presented in the manuscript, pose a threat to:

No	Yes	
☒	☐	Public health
☒	☐	National security
☒	☐	Crops and/or livestock
☒	☐	Ecosystems
☒	☐	Any other significant area

Experiments of concern

Does the work involve any of these experiments of concern:

No	Yes	
☒	☐	Demonstrate how to render a vaccine ineffective
☒	☐	Confer resistance to therapeutically useful antibiotics or antiviral agents
☒	☐	Enhance the virulence of a pathogen or render a nonpathogen virulent
☒	☐	Increase transmissibility of a pathogen
☒	☐	Alter the host range of a pathogen
☒	☐	Enable evasion of diagnostic/detection modalities
☒	☐	Enable the weaponization of a biological agent or toxin
☒	☐	Any other potentially harmful combination of experiments and agents

Plants

Seed stocks

Report on the source of all seed stocks or other plant material used. If applicable, state the seed stock centre and catalogue number. If plant specimens were collected from the field, describe the collection location, date and sampling procedures.

Novel plant genotypes

Describe the methods by which all novel plant genotypes were produced. This includes those generated by transgenic approaches, gene editing, chemical/radiation-based mutagenesis and hybridization. For transgenic lines, describe the transformation method, the number of independent lines analyzed and the generation upon which experiments were performed. For gene-edited lines, describe the editor used, the endogenous sequence targeted for editing, the targeting guide RNA sequence (if applicable) and how the editor was applied.

Authentication

Describe any authentication procedures for each seed stock used or novel genotype generated. Describe any experiments used to assess the effect of a mutation and, where applicable, how potential secondary effects (e.g. second site T-DNA insertions, mosiacism, off-target gene editing) were examined.

ChIP-seq

Data deposition

☐ Confirm that both raw and final processed data have been deposited in a public database such as GEO.

☐ Confirm that you have deposited or provided access to graph files (e.g. BED files) for the called peaks.

Data access links
May remain private before publication.

For "Initial submission" or "Revised version" documents, provide reviewer access links. For your "Final submission" document, provide a link to the deposited data.

Files in database submission

Provide a list of all files available in the database submission.

Genome browser session
(e.g. UCSC)

Provide a link to an anonymized genome browser session for "Initial submission" and "Revised version" documents only, to enable peer review. Write "no longer applicable" for "Final submission" documents.

Methodology

Replicates

Describe the experimental replicates, specifying number, type and replicate agreement.

Sequencing depth

Describe the sequencing depth for each experiment, providing the total number of reads, uniquely mapped reads, length of reads and whether they were paired- or single-end.

Antibodies

Describe the antibodies used for the ChIP-seq experiments; as applicable, provide supplier name, catalog number, clone name, and lot number.

Peak calling parameters

Specify the command line program and parameters used for read mapping and peak calling, including the ChIP, control and index files used.

Data quality

Describe the methods used to ensure data quality in full detail, including how many peaks are at FDR 5% and above 5-fold enrichment.

Software

Describe the software used to collect and analyze the ChIP-seq data. For custom code that has been deposited into a community repository, provide accession details.

Flow Cytometry

Plots

Confirm that:

☒ The axis labels state the marker and fluorochrome used (e.g. CD4-FITC).

☒ The axis scales are clearly visible. Include numbers along axes only for bottom left plot of group (a 'group' is an analysis of identical markers).

☒ All plots are contour plots with outliers or pseudocolor plots.

☒ A numerical value for number of cells or percentage (with statistics) is provided.

Methodology

Sample preparation	Shaved back skin (approximately 2 cm2 containing both injection sites) was cut into small pieces and digested with collagenase IV (C1889; Sigma-Aldrich), 0.166 mg/ml hyaluronidase (H3506; Sigma-Aldrich) and 0.1 mg/ml DNase I (11284932001; Merck) for 1 hour under constant shaking at 37°C.<br>Skin-draining lymph nodes were incubated in for 20 minutes at 37°C with 2.4 mg/ml collagenase IV (C1889; Sigma-Aldrich) and 2.4 mg/ml DNase I (11284932001; Merck).<br>After tissue digestion, cells were filtered through a 100um cell strainer.
Instrument	Data was acquired using a BD FACSCanto II or a CytoFLEX flow cytometer (Beckman-Coulter, Brea, California, USA).
Software	Data was analysed with CytExpert (Beckman-Coulter) or FlowJo (BD).
Cell population abundance	As described in the manuscript.
Gating strategy	As described in the manuscript.

☒ Tick this box to confirm that a figure exemplifying the gating strategy is provided in the Supplementary Information.

Magnetic resonance imaging

Experimental design

Design type	*Indicate task or resting state; event-related or block design.*
Design specifications	*Specify the number of blocks, trials or experimental units per session and/or subject, and specify the length of each trial or block (if trials are blocked) and interval between trials.*
Behavioral performance measures	*State number and/or type of variables recorded (e.g. correct button press, response time) and what statistics were used to establish that the subjects were performing the task as expected (e.g. mean, range, and/or standard deviation across subjects).*

Acquisition

Imaging type(s)	*Specify: functional, structural, diffusion, perfusion.*
Field strength	*Specify in Tesla*
Sequence & imaging parameters	*Specify the pulse sequence type (gradient echo, spin echo, etc.), imaging type (EPI, spiral, etc.), field of view, matrix size, slice thickness, orientation and TE/TR/flip angle.*
Area of acquisition	*State whether a whole brain scan was used OR define the area of acquisition, describing how the region was determined.*

Diffusion MRI ☐ Used ☐ Not used

Preprocessing

Preprocessing software	*Provide detail on software version and revision number and on specific parameters (model/functions, brain extraction, segmentation, smoothing kernel size, etc.).*
Normalization	*If data were normalized/standardized, describe the approach(es): specify linear or non-linear and define image types used for transformation OR indicate that data were not normalized and explain rationale for lack of normalization.*

Normalization template

Describe the template used for normalization/transformation, specifying subject space or group standardized space (e.g. original Talairach, MNI305, ICBM152) OR indicate that the data were not normalized.

Noise and artifact removal

Describe your procedure(s) for artifact and structured noise removal, specifying motion parameters, tissue signals and physiological signals (heart rate, respiration).

Volume censoring

Define your software and/or method and criteria for volume censoring, and state the extent of such censoring.

Statistical modeling & inference

Model type and settings

Specify type (mass univariate, multivariate, RSA, predictive, etc.) and describe essential details of the model at the first and second levels (e.g. fixed, random or mixed effects; drift or auto-correlation).

Effect(s) tested

Define precise effect in terms of the task or stimulus conditions instead of psychological concepts and indicate whether ANOVA or factorial designs were used.

Specify type of analysis: ☐ Whole brain ☐ ROI-based ☐ Both

Statistic type for inference

(See Eklund et al. 2016)

Specify voxel-wise or cluster-wise and report all relevant parameters for cluster-wise methods.

Correction

Describe the type of correction and how it is obtained for multiple comparisons (e.g. FWE, FDR, permutation or Monte Carlo).

Models & analysis

n/a	Involved in the study
☐	☐ Functional and/or effective connectivity
☐	☐ Graph analysis
☐	☐ Multivariate modeling or predictive analysis

Functional and/or effective connectivity

Report the measures of dependence used and the model details (e.g. Pearson correlation, partial correlation, mutual information).

Graph analysis

Report the dependent variable and connectivity measure, specifying weighted graph or binarized graph, subject- or group-level, and the global and/or node summaries used (e.g. clustering coefficient, efficiency, etc.).

Multivariate modeling and predictive analysis

Specify independent variables, features extraction and dimension reduction, model, training and evaluation metrics.

This checklist template is licensed under a Creative Commons Attribution 4.0 International License, which permits use, sharing, adaptation, distribution and reproduction in any medium or format, as long as you give appropriate credit to the original author(s) and the source, provide a link to the Creative Commons license, and indicate if changes were made. The images or other third party material in this article are included in the article's Creative Commons license, unless indicated otherwise in a credit line to the material. If material is not included in the article's Creative Commons license and your intended use is not permitted by statutory regulation or exceeds the permitted use, you will need to obtain permission directly from the copyright holder. To view a copy of this license, visit http://creativecommons.org/licenses/by/4.0/
